# Supplementary material for: A strength inversion origin for non-volcanic tremor
Source: Nat Commun. 2022 Apr 28;13:2311. doi: 10.1038/s41467-022-29944-8 (PMC9050742; doi:10.1038/s41467-022-29944-8)
Supplement: Supplementary file 1 — Supplementary Info [file 41467_2022_29944_MOESM1_ESM.pdf]

A strength inversion origin for non-volcanic tremor

Paola Vannucchi[1], Alexander Clarke[2], Albert de Montserrat[3], Audrey Ougier-Simonin[4], Luca Aldega[5], Jason P. Morgan*[6]

[1] Universitá degli Studi di Firenze, Firenze 50121, Italy
[2] University College London, Gower Street, London WC1E 6BT, UK
[3] Universitá degli Studi di Padova, Padova 35131, Italy
[4] British Geological Survey, Keyworth NG12 5GG, UK
[5] Universitá di Roma, La Sapienza, Roma 00185, Italy
[6] SUSTech, Shenzhen 518000, China

* Corresponding Author

Tables

Table S1: Glossary of Rheological Terms for Geology and Continuum Mechanics

**Geology**			**Continuum Mechanics**		
**Term**	**Definition**	**Usage**	**Term**	**Definition**	**Usage**
Strength [18]	Resistance to permanent deformation by *either* **flow** *or* **failure** [18].	A relative term except for Mohr-Coulomb failure where referred to as 'yield strength'. Corresponding continuum mech. terms: *more viscous* (**flow**) or *tougher* (**failure**)	*Toughness*	Energy that can be absorbed (per unit volume) by a material prior to its **failure**	Measure of the body's resistance to failure by fracture when a body is stressed
			Critical Stress Intensity Factor	K_c (**failure** property)	In fracture mechanics, if a crack's stress intensity factor $K>K_c$, the crack will grow
Competence [55]	**Flow** or **Failure** Property [55]	More Competent ≈ either more viscous (stiffer) or breaks more readily	*Viscosity*	Scaling between stress and strain-rate tensors for a **viscous fluid**	Used to determine viscous creep rates in response to loads
Stiffness	**Flow** property	Stiffer ≈ More Viscous	S*tiffness* (elastic)	Coefficients scaling **elastic** stress tensor to strain tensor	Used to determine elastic strains in response to loads
Cohesion "c"	Constant term (i.e. non-σ_n -dependent term) in Mohr-Coulomb **failure**	Brittle shear yield strength $\tau = c+A\sigma_n$. If the shear stress reaches the shear yield strength, failure will occur.	*Cohesion*	Constant term (i.e. non-σ_n -dependent term) in generalized **failure**	e.g. Drucker-Prager Yield Criterion (Simplest σ_n -dependent form of plastic yielding). The Drucker-Prager yield surface is a smooth version of the Mohr-Coulomb yield surface.

τ = Brittle shear yield strength along a surface

σ_n = Normal stress (positive in compression for geology, negative in compression for continuum mechanics)

A = Constant that is usually written as tan(ϕ), where ϕ is the internal angle of friction

Supplementary Information on the Osa Melange

The section of the Osa Melange outcropping in the northwest part of the Osa Peninsula is known as the San Pedrillo Unit (Buchs et al., 2009; Clarke et al., 2018; Di Marco et al., 1995; Vannucchi et al., 2006). This unit represents the oldest member of the Osa Melange with depositional ages from the Campanian to the middle Eocene(Di Marco et al., 1995), while its involvement in the subduction zone deformation is tentatively dated to the late Oligocene-early Miocene (Vannucchi et al., 2006). The San Pedrillo Unit is composed of a volcanoclastic greywacke matrix containing blocks of basalt, gabbro, turbidites, hemi-pelagic and pelagic sediments, and subordinate dolerite(Buchs et al., 2009; Clarke et al., 2018; Meschede et al., 1999; Vannucchi et al., 2006). Both the blocks and the matrix show widespread, but not uniform low-grade metamorphic alteration with hydrous minerals precipitation not exceeding the prehnite-pumpellyite facies (SM Figure 1) (Buchs et al., 2009; Meschede et al., 1999; Vannucchi et al., 2006). Zeolites, mostly occurring in veins, are prevalent, but clay minerals, chlorite, epidote, calcite, hematite and pyrite are also common both in veins and replacing phenocrystals, groundmass and mineral grains in the sediments. This mineralogical association defines a metamorphic environment with a maximum temperature of ~250°C. This temperature is further confirmed by the twin set types in the calcite crystals (Meschede et al., 1999). The mélange matrix also contains smectites; the presence of smectite helps to attribute the low-grade metamorphism to a pre-subduction hydrothermal ocean-floor alteration. In fact, since the onset of illitization and/or chloritization of smectite during shallow subduction occurs at 60°C and in general continues to temperatures as high as 150°C (Moore and Saffer, 2001; Saffer et al., 2008; Środoń, 1999), prograde metamorphism during subduction to the prehite/pumpellyite facies is incompatible with its presence.

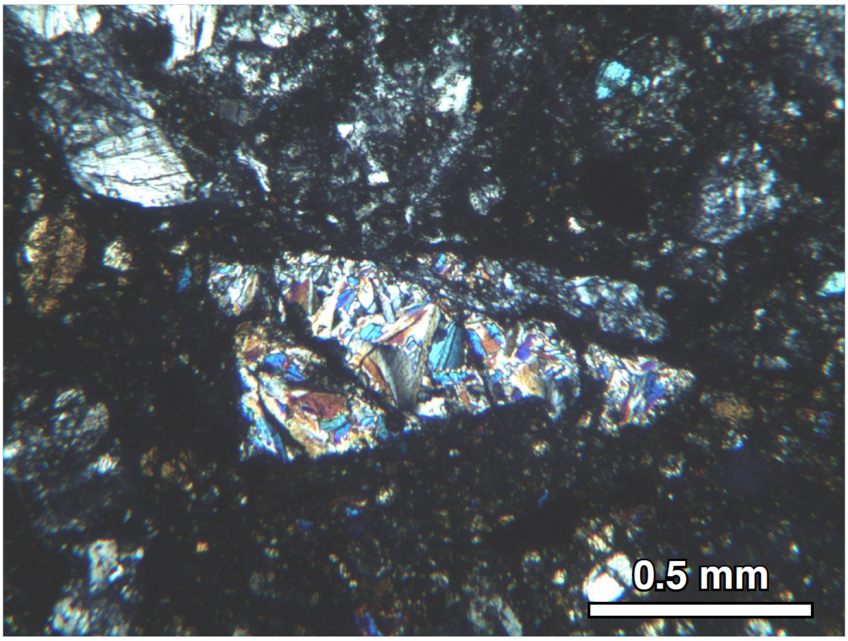

Supplementary Material Figure 1: Petrographic photomicrograph in cross-polarized light of prehnite in volcanoclastic matrix.

Based on the relative abundance of the different lithological components the San Pedrillo Unit has been subdivided in packages by Clarke et al (2018). Although the deformation style of the San Pedrillo Unit does not change throughout the different packages, the samples and the figures presented in this paper are coming from the ~1km-thick Punta Marenco Package, where basaltic blocks are embedded in the volcanoclastic matrix. This is a somewhat simple system within the melange when compared to the other packages.

The Osa Mélange basalt blocks are mainly composed of plagioclase lathes with clinopyroxene and subordinate olivine (SM Figure 2). Folded veinlets of zeolites are widespread. XRD analysis on the basalt sample tested in the triaxial experiments reveal the presence of augite, Ca-plagioclase, zeolites such as analcime, thomsonite and laumontite, chlorite, celadonite and traces of quartz (SM Figure 3).

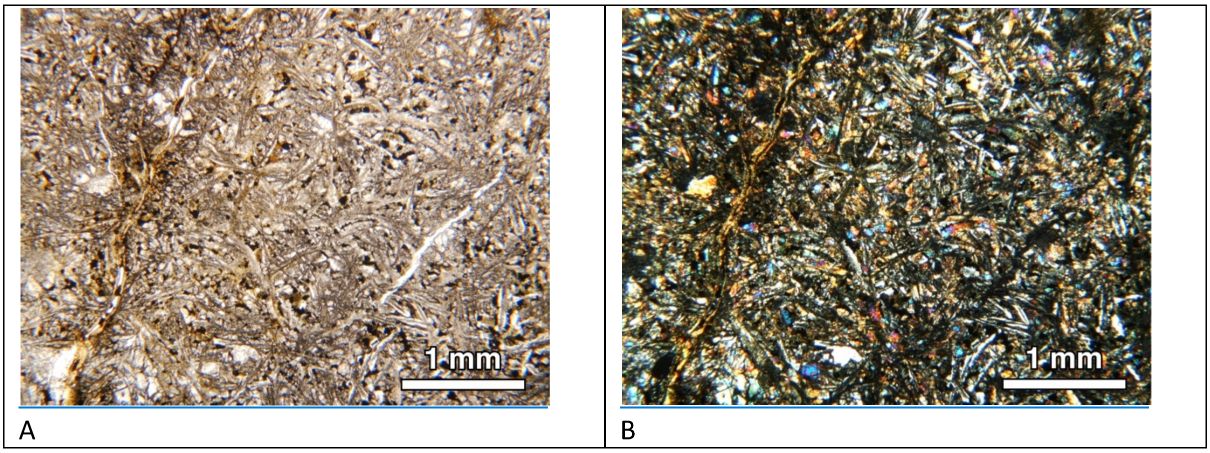

Supplementary Material Figure 2 Petrographic microphotographs in plane- (A) and crossed-polarized light (B) of Osa Melange basalt.

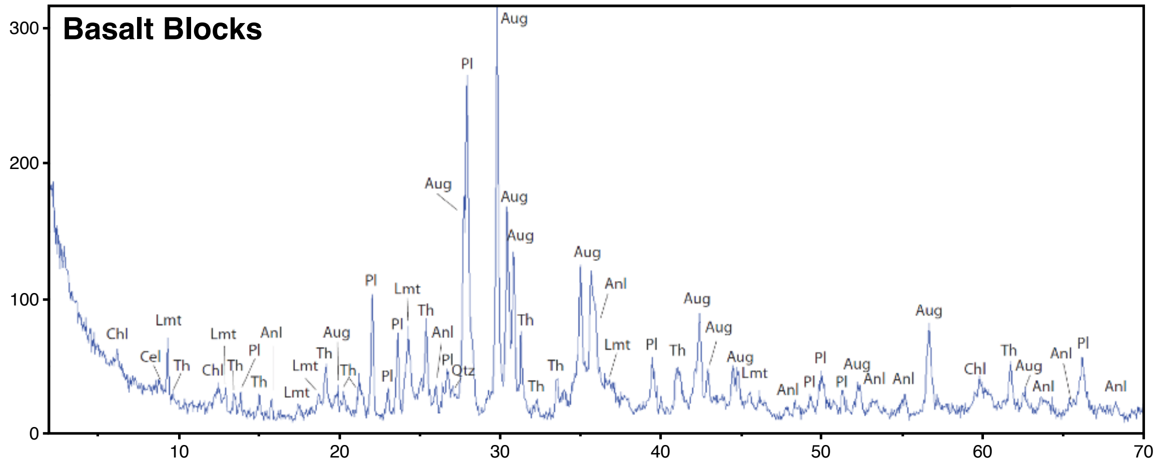

Supplementary Material Figure 3. XRD pattern of Osa Melange basalt tested in triaxial experiments (N 8 ° 41’ 20.26”; W 83 ° 42’ 16.81”). Chl: chlorite; Cel: celadonite; Lmt: laumontite; Th: thomsonite; Anl: analcime; Pl: Ca-plagioclase; Aug: augite; Qtz: quartz.

The volcanoclastic matrix is mostly composed of clays, rounded silt-sized pyroxene and plagioclase grains, and basalt lithic clasts (SM Figure 4). Sparse microfossils, mostly radiolarians, are also present. Folded veinlets of zeolites and calcite are widespread. From XRD analysis of the sample of volcanoclastic matrix tested in the triaxial experiments, the matrix contains Ca-plagioclase and variably altered clinopyroxene, kaliophilite and low-temperature hydrous zeolite minerals such as analcime, thomsonite and laumontite, chlorite, smectite, and celadonite (SM Figure 5). This mineralogical assemblage is similar to the bulk composition of the basalt sample, even though an increased amount of quartz (SM Figure 5) agrees with some recrystallized radiolarians observed in thin section. This result further confirms that this sediment is directly derived from the erosion of similar basalt (Clarke et al., 2018).

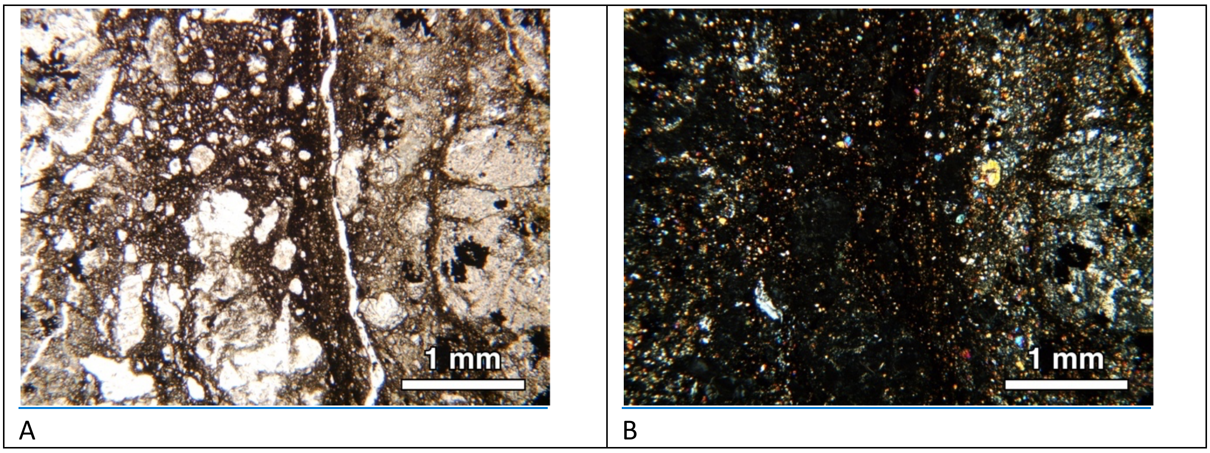

Supplementary Material Figure 4: Petrographic microphotographs in plane- (A) and crossed-polarized light (B) of Osa Melange volcanoclastic matrix.

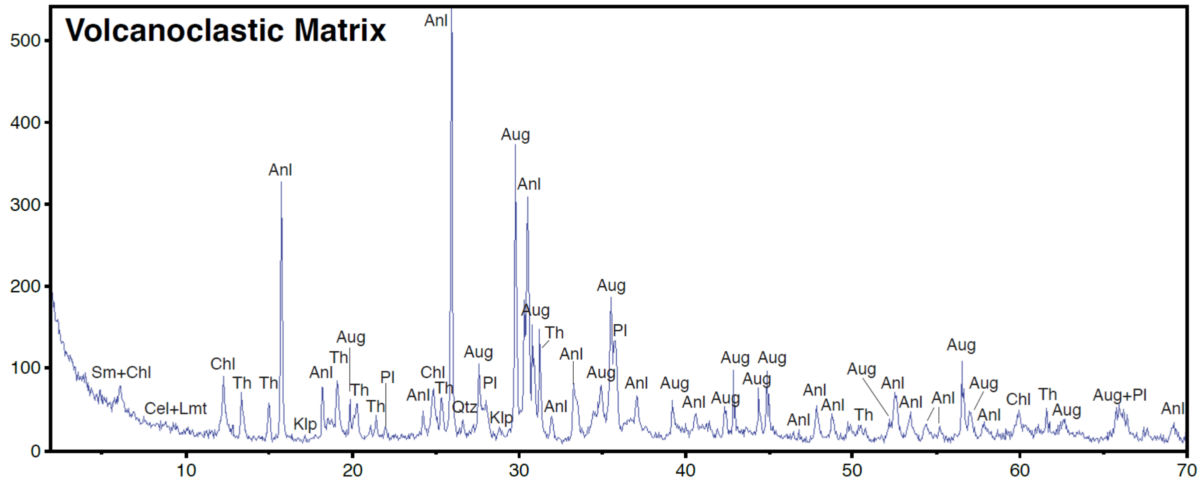

Supplementary Material Figure 5: XRD pattern of Osa Melange matrix from sample tested in triaxial experiments (N 8°41' 23.9", W 83°42' 10.7"). Sm: smectite; Chl: chlorite; Cel: celadonite; Lmt: laumontite; Th: thomsonite; Anl: analcime; Pl: Ca-plagioclase; Aug: augite; Klp: kaliophilite; Qtz: quartz.

Exhumation of the Osa Melange

A complete analysis of the forearc deformation responsible for the exhumation of the Osa Melange is given in Sak et al. (2004), Vannucchi et al. (2006), and Morell et al. (2019). All authors agree that the post-subduction deformation of the Osa Melange is characterised by steeply dipping to subvertical brittle fractures and faults that cut through the matrix and the blocks. These fractures and faults are related to vertical tectonism inferred to be induced by the subduction of bathymetric relief on the downgoing plate.

Supplementary Numerical Experiments

In the main text we discuss the results and implications of a suite of numerical experiments in which the blocks in a shear channel were idealized as a set of five alternating en-echelon elliptical bodies. After some experimentation, the idealizations in these experiments were chosen to allow us to isolate the origins of observed mechanical behaviour. Here we present and discuss a further suite of experiments that were performed to further test that our findings are not the byproduct of our intentionally simplified experimental configuration. The experiments shown here focus on assessing the degree that observed strong-block-in-weak-matrix behaviour and weak-block-in-strong-matrix behaviour vary with rheological and geometric parameter variations, and with the volume fraction of block material within the shear channel.

Supplementary Figure 6 shows four additional experiments on configurations with high viscosity (>=1e19 Pa-s) competent (cohesion=20 MPa) blocks in a lower viscosity, equally competent matrix. (Supplement Table 1 defines the sometimes contrasting geological and engineering continuum mechanics terminology used to describe rheological properties.) Panels A-B show the shear-stress (A) and ongoing plastic failure(B) of a similar but more complex scenario than that analyzed in the

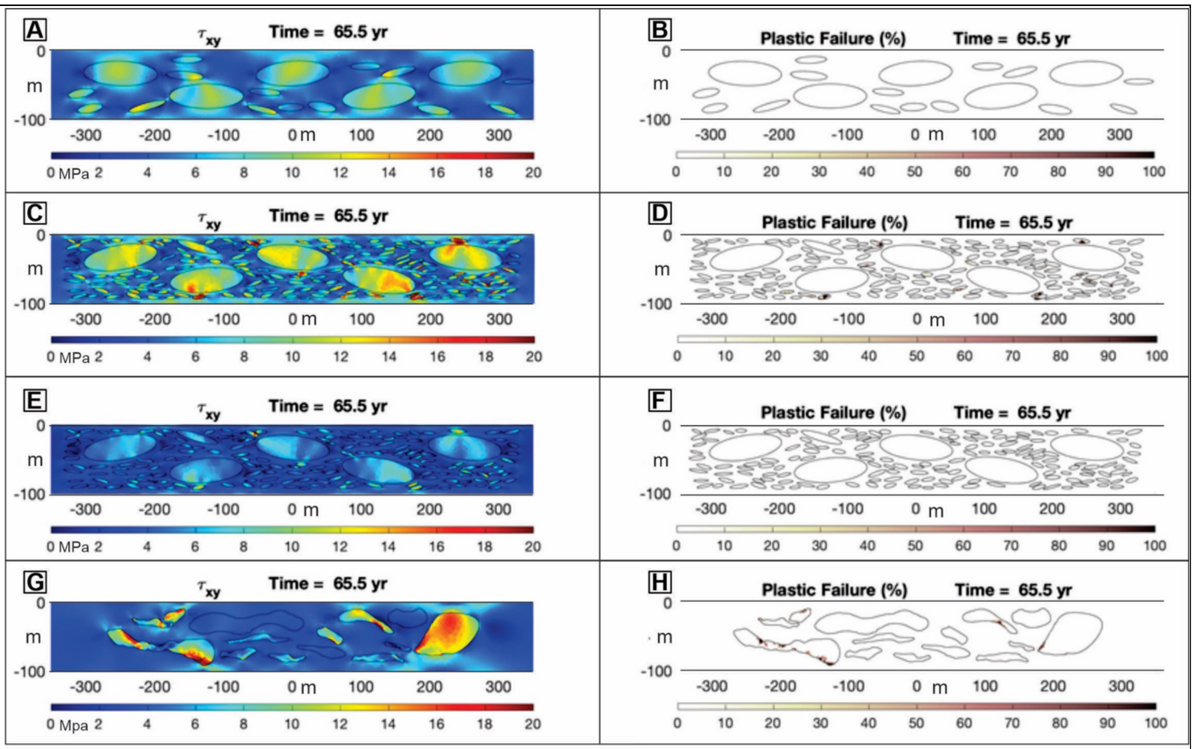

Supplementary Material Figure 6. Shear channel with high viscosity (>=10^{19} Pa-s) and competent (cohesion=20 MPa) blocks in a lower viscosity (10^{18} Pa-s), equally competent matrix. Each pair of panels shows the shear stress field and places where blocks are failing for a range of block-size distributions. Panels (A,B) – block fraction = 34% and block viscosity = 10^{20} Pa-s. No blocks fail. A visually identical stress pattern is seen for any block viscosity >10^{19} Pa-s — the channel stress distribution is insensitive to block viscosity as long as it is at least an order of magnitude greater than the matrix viscosity. Panels (C,D) – block fraction = 44%. Here, a few of the thinnest blocks fail under the higher channel stresses induced by the greater block fraction. Larger blocks do not fail, only small thin blocks that are in general close to either the (artificially rigid) channel walls or to large blocks. This failure pattern would not lead to tremor sources the sizes of the larger blocks. Panels (E,F) – block fraction = 44%. Here, channel viscosity is reduced by a factor of 2 to 5x10^{17} Pa-s in comparison to the otherwise identical experiment shown in panels (C,D). No blocks fail. Panels (G,H) This experiment uses the block geometry mapped by Clarke et al. (2018). In this case, the thinnest parts of some blocks (and one large block corner-tip) fail under the ambient channel stresses associated with a 10^{18} Pa-s channel viscosity. When failure occurs, it also takes place at isolated discrete locations, which would not correspond to tremor sources with sizes characteristic of the size of the larger blocks.

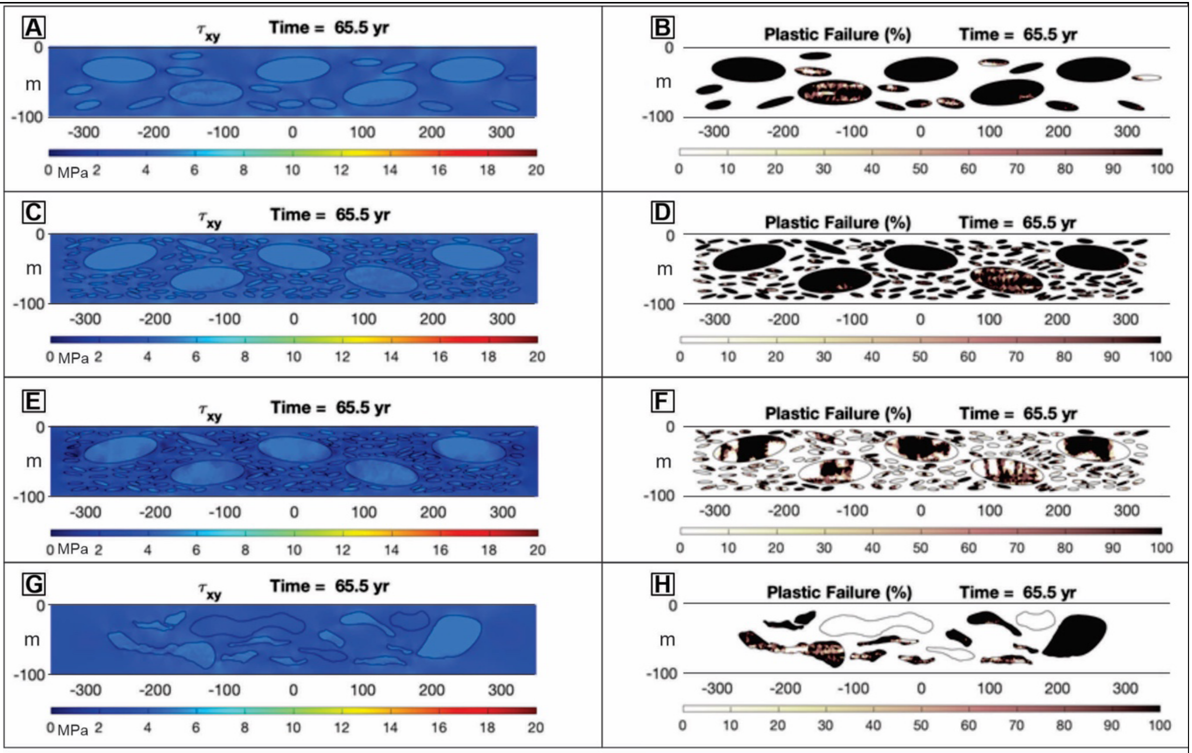

Supplementary Material Figure 7. Shear channel with high viscosity(>=10^{19} Pa-s) but less competent (cohesion=5 MPa) blocks embedded in a lower viscosity (1e18 Pa-s), higher competency (cohesion = 20MPa) matrix. Each pair of panels shows the shear stress field and places where blocks are failing for a range of block-size distributions. Panels (A,B) – block fraction = 34% and block viscosity = 10^{20} Pa-s. As in the previous examples, the channel stress distribution is insensitive to block viscosity as long as it is at least an order of magnitude greater than the matrix viscosity. Once stresses in a block exceed the block's cohesion, it behaves as a weaker material irrespective of its nominal viscosity. All larger blocks, and almost all smaller blocks fail plastically, with implied stress drops and failure regions characteristic of observed tremor. Panels (C,D) – block fraction = 44%. Again, all blocks fail plastically. Panels (E,F) – block fraction = 44%. Matrix viscosity lowered to 5x1017 Pa-s. Even in this lower viscosity shear channel, the larger blocks all fail plastically and generate block-crossing regions of failure, with implied stress drops and failure geometries compatible with seismic observations of tremor. Panels (G,H) The block geometry mapped by Clarke et al. (2018). Once again, larger blocks fail in through-going regions of failure. More complex behaviour (e.g. stress-shadowing which 'protects' some blocks from reaching their failure stress) is also seen.

main text. The material properties are the same as in the primary experiments, but here the five strong blocks have small random perturbations to their orientations, while being surrounded by a suite of quasi-randomly distributed smaller blocks with the same overall preferred orientation and identical material properties. Block material fills 34% of the channel. In this case, channel shear stresses are slightly more elevated (due to the higher material fraction of more viscous block material), while continuing to exhibit factor-of-two stress concentrations in blocks, in particular within the larger blocks (panel A). No blocks fail (panel B) — the key behaviour noted in the main text. Note that a visually identical stress pattern will be seen for any block viscosity >1e19 Pa-s — e.g. the channel stress distribution is insensitive to block viscosity as long as it is at least an order of magnitude greater than the matrix viscosity. In these experiments, the matrix between two adjacent blocks often has a higher-than-average stress as it resists being squeezed out from between the two blocks. This behaviour is well-captured by the Lagrangian deformation approximation used here, but is not captured by improperly resolved particle-in-cell treatments of block and matrix rheology, a numerical algorithm that has been commonly applied in related numerical modelling (cf. Beall et al., 2019). Panels C and D show an example with an even higher overall block fraction of 44%. In this

case, a few of the thinnest blocks fail under the higher channel stresses induced by the greater block fraction. In general, the first blocks to fail in this and a suite of similar random blob experiments are the thinnest, most elongated blocks, suggesting that failure is shaped by flexural stresses in the blocks. (This is a useful topic for future study). Note that the larger blocks in panel D do not fail, only small thin blocks that are in general close to either the channel walls or to large blocks. This failure pattern would not lead to tremor sources with sizes characteristic of the larger blocks. In panels E and F the matrix viscosity is reduced by a factor of two in comparison to the other examples in this figure, to $5x10^{17}$ Pa-s. In this case, channel and block stresses are correspondingly reduced, and no blocks fail. Finally, panels G and H show an example based on the geometry mapped by Clarke et al. (2018). In this case, the thinnest parts of some blocks, and one corner of a larger more equant block, do fail under the ambient channel stresses associated with a 10^{18} Pa-s shear channel viscosity. When failure occurs, it takes place at isolated point-like locations, which would not correspond to tremor sources with sizes characteristic of the size of the larger blocks, as is inferred from seismic observations. These examples corroborate this paper's finding that competent high-viscosity blocks in a shear channel will not produce the failure patterns characteristic of observed seismic tremor.

Supplementary Figure 7 shows the behaviour of the same examples shown in the previous figure for the case where the block-cohesion is reduced to 5 MPa, e.g. with the same mechanical weakness of blocks relative to matrix as that explored in the examples discussed in the main text. In all examples (Panels 7B,D,F,H) large volumes of the blocks fail plastically, with implied stress drops and failure regions characteristic of seismically observed tremor. Even when the matrix viscosity is reduced to $5x10^{17}$ Pa-s (Panel 7F), the large blocks still fail in through-going regions. Thinner, more lathe-like blocks fail more easily than thicker blocks. Blocks typically fail after less than 5-10 years of shear-loading (not shown here, we chose to show the same 65.25 year timestep for all model runs). Other phenomena are also evident. For example, sometimes blocks will experience lower stresses and less failure due to stress 'shadowing' by adjacent blocks (see panel B and panel H in particular). These additional experiments lend further support to the paper's finding that weak low-cohesion blocks can produce failure patterns characteristic of observed seismic tremor.

References

Buchs, D. M., Baumgartner, P. O., Baumgartner-Mora, C., Bandini, A. N., Jackett, S. J., Diserens, M. O., and Stucki, J., 2009, Late Cretaceous to Miocene seamount accretion and melange formation in the Osa and Burica Peninsulas (Southern Costa Rica): episodic growth of a convergent margin, *in* James, K. H., Lorente, M. A., and Pindell, J. L., eds., Origin and Evolution of the Caribbean Plate, Volume 328, p. 411-456.

Clarke, A. P., Vannucchi, P., and Morgan, J., 2018, Seamount chain-subduction zone interactions: Implications for accretionary and erosive subduction zone behavior: Geology, v. 46, no. 4, p. 367-370.

Di Marco, G., Baumgartner, P. O., and Channell, J. E. T., 1995, Late Cretaceous-early Tertiary paleomagnetic data and revised tectonostratigraphic subdivision of Costa Rica and western Panama, *in* Mann, P., ed., Geologic and Tectonic Development of the Caribbean Plate Boundary in Southern Central America, Volume 295: Boulder, Colorado, Geological Society of America Special Paper, p. 1-27.

Meschede, M., Zweigel, P., Frisch, W., and Volker, D., 1999, Melange formation by subduction erosion: the case of the Osa melange in southern Costa Rica: Terra Nova, v. 11, no. 4, p. 141-148.

Moore, J. C., and Saffer, D., 2001, Updip limit of the seismogenic zone beneath the accretionary prism of southwest Japan: An effect of diagenetic to low-grade metamorphic processes and increasing effective stress: Geology, v. 29, no. 2, p. 183-186.
Morell, K. D., Fisher, D. M., & Bangs, N. (2019). Plio-Quaternary outer forearc deformation and mass balance of the southern Costa Rica convergent margin. Journal of Geophysical Research: Solid Earth, 124(9), 9795-9815.
Saffer, D. M., Underwood, M. B., and McKiernan, A. W., 2008, Evaluation of factors controlling smectite transformation and fluid production in subduction zones: Application to the Nankai Trough,: Island Arc, v. 17, p. 208-230.
Sak, P. B.,D.M. Fisher, and T.W.Gardner (2004), Effects of subducting seafloor roughness on upper plate vertical tectonism: Osa Peninsula, Costa Rica, Tectonics, 23, TC1017, doi:10.1029/2002TC001474.
Środoń, J., 1999, Nature of mixed-layer clays and mechanisms of their formation and alteration: Annual Review of Earth and Planetary Sciences, v. 27, p. 19-53.
Vannucchi, P., Fisher, D. M., Gardner, T. W., and Bier, S., 2006, From seamount accretion to tectonic erosion: Formation of Osa Mélange and the effects of Cocos Ridge subduction in southern Costa Rica: Tectonics, v. 25, no. TC2004.
